# Preliminary experience with biodegradable implants for fracture fixation

**DOI:** 10.4103/0019-5413.41856

**Published:** 2008

**Authors:** Mandeep S Dhillon, Sharad Prabhakar, Chandiralingam Prasanna

**Affiliations:** Department of Orthopedic Surgery, Post Graduate Institute of Medical Education and Research, Chandigarh, India

**Keywords:** Biodegradable implants, fractures, surgeon confidence

## Abstract

**Background:**

Biodegradable implants were designed to overcome the disadvantages of metal-based internal fixation devices. Although they have been in use for four decades internationally, many surgeons in India continue to be skeptical about the mechanical strength of biodegradable implants, hence this study.

**Materials and Methods:**

A prospective study was done to assess the feasibility and surgeon confidence level with biodegradable implants over a 12-month period in an Indian hospital. Fifteen fractures (intra-articular, metaphyseal or small bone fractures) were fixed with biodegradable implants. The surgeries were randomly scheduled so that different surgeons with different levels of experience could use the implants for fixation.

**Results:**

Three fractures (one humeral condyle, two capitulum), were supplemented by additional K-wires fixation. Trans-articular fixator was applied in two distal radius and two pilon fractures where bio-pins alone were used. All fractures united, but in two cases the fracture displaced partially during the healing phase; one fibula due to early walking, and one radius was deemed unstable even after bio-pin and external fixator.

**Conclusions:**

Biodegradable -implants are excellent for carefully selected cases of intra-articular fractures and some small bone fractures. However, limitations for use in long bone fractures persist and no great advantage is gained if a “hybrid” composite is employed. The mechanical properties of biopins and screws in isolation are perceived to be inferior to those of conventional metal implants, leading to low confidence levels regarding the stability of reduced fractures; these implants should be used predominantly in fracture patterns in which internal fixation is subjected to minimal stress.

## INTRODUCTION

Biodegradable implants have evolved over the last four decades from simple sheets or films of polymers suitable for experimental use to implants of more complex design, such as biodegradable pins, wires and plates for internal fixation of fractures. They were designed to overcome the disadvantages of metal-based internal fixation devices such as stress shielding,^1^ corrosion,^2^ accumulation of metal in tissues,^3^ titanium hypersensitivity,^4^ pain,^5^ interference in radiological studies^6^ and need of a second surgery for implant removal. Despite this progress, these materials are still only used in small numbers, and the scope of their application has been limited. Although many of the principles of metallic internal fixation also apply to absorbable internal fixation, significant differences exist as well.^7^ Many surgeons are unfamiliar with biodegradable implant technology and hence poses an element of doubt (low confidence level) regarding their mechanical strength as compared to metallic implants, at least during the initial part of their usage.

Keeping this in mind, we planned a prospective study of fixation of intra-articular, metaphyseal fracture of long bone and small bone fractures with biodegradable implants to assess the outcome of surgery and surgeon confidence level in the usage of these implants.

## MATERIALS AND METHODS

A prospective study was conducted in 15 patients who presented with fifteen metaphyseal, intra-articular or small bone (hand and foot) fractures, to the emergency department. Five orthopedic trauma surgeons comprising one senior consultant, two junior consultants and two registrars were assessed for their confidence levels with the biodegradable implants.

Inclusion criteria were closed fractures, intra-articular or long bone metaphyseal fractures, seen at ages younger than 55 years. The fracture site and pattern was evaluated, and appropriate surgical plan including the type of implant that was to be used was decided upon (plates, screws, pins, etc). All types of implants (INION, Finland) which could potentially be used were kept ready before the surgery.

In fractures where biodegradable pins were used the fracture fragment was anatomically reduced and a K-wire of either 1.5 mm or 2 mm was drilled to appropriate length, the fracture reduction and the position of the wire were confirmed by image intensifier, and depth determined by measuring size of K-wire outside. The wire was then removed and the drill hole was flushed to remove any bone debris and the biodegradable implant of the same size as the K-wire (1.5 or 2 mm) was inserted into the channel by gentle tapping using the applicator. Protruding pin was cut flush with the bone surface. For screw placement, a drill hole was made using the appropriate drill bit and then tapped. The screws were placed using the specially designed torque-limiting screwdrivers to prevent screw head breakage. Biodegradable plating techniques were similar to standard plating methods; the plates were molded according to the bone surface after pre-treatment in the water bath provided (at 70°C), and applied to the well-reduced and stabilized fracture.

Any intra-operative difficulties with bio-implant use were noted and recorded; the degree of anatomical reduction and stability of fracture fixation were judged intra-operatively by the operating surgeon. Randomization of the operating surgeon was done by indirect methods, as all cases were operated in the emergency department, and the duty roster decided who would be operating on which case. Each surgeon was asked specific questions after the procedure about his confidence level with the implant and the need to supplement fixation with conventional implants. If supplementation was needed, his comments and reason for employing supplemental fixation were recorded. Postoperative protocol for all operated cases was the same as with conventional implants, except in ankle fractures, where patients were asked to delay more than 50% weight-bearing till evidence of fracture healing. The cost of the implant varied; biopins cost Rs. 7000 for a pack of three pins, bioscrews cost Rs 2000 for a set of two screws and a six-hole biodegradable plate cost Rs. 7000.

## RESULTS

**Figure 1:**
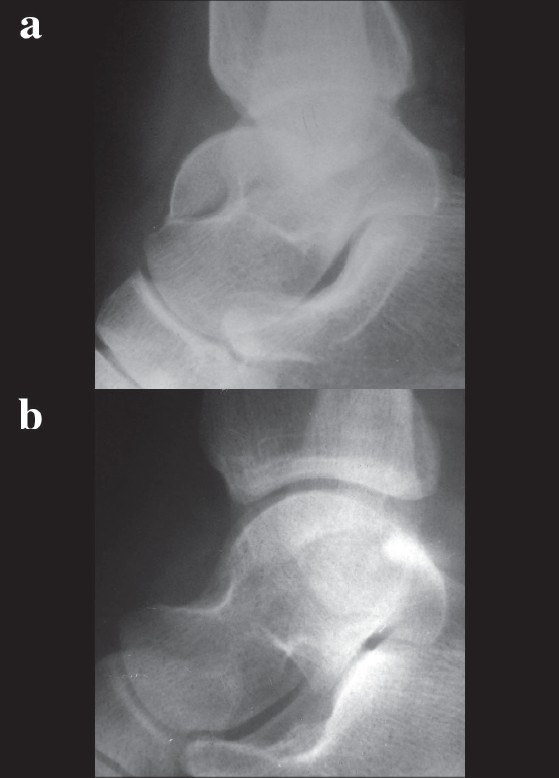
(a) Preoperative X-ray showing fracture talus. (b) 6 months postoperative X-ray shows fracture talus fixed with bio-screws showing excellent reduction and fracture union

**Figure 2:**
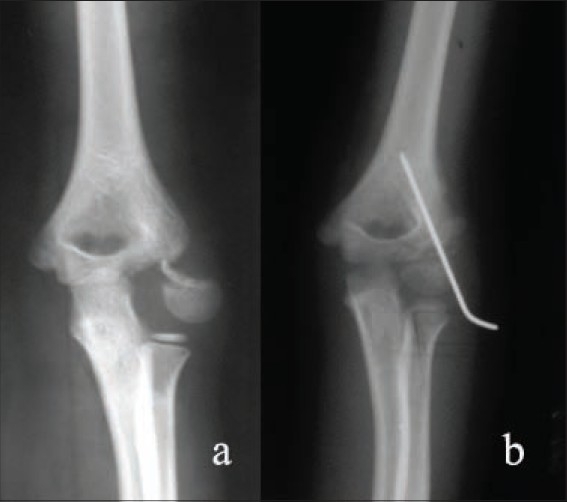
(a) Preoperative X-ray (anteroposterior view) of left elbow showing fracture lateral condyle of humerus, (b) Postoperative X-ray (anteroposterior view) of left elbow shows fracture fixed with bio-pin and additional K-wire because of stability concerns. The patient had full range of motion

A total of 15 patients with eight male and seven female were included in the study. The age range was 7-54 years with a mean age of 30.1 years. The mode of injury was a roadside accident (n=7) and a fall from height/at play (n=8). Right side involvement was seen in seven fractures and eight had left side involvement. The upper limb was involved in eight cases and the lower limb in seven cases. The fracture distribution was as follows: supracondylar humerus (2), intercondylar humerus (1), lateral condyle humerus (1), capitulum (2), distal radius (2), bimalleolar ankle fracture (1), trimalleolar ankle fracture (1), unimalleolar ankle fracture (1), pilon fractures (2) and fracture neck of talus (2). Excellent anatomic reduction was achieved in 73.3% and fair reduction in 26.7% cases [Figure 1]. The intra-operative stability was excellent in 66.7% and good in 33.3% as assessed by the operating surgeon. The cases of supracondylar (2), intercondylar (1), lateral condyle humerus (1) fractures and distal radius fractures (2) were stabilized with bio-pins. The unimalleolar lateral malleolus fracture was fixed with a six-hole biodegradable plate. The lateral malleolar component of the bimalleolar fracture was also fixed with a biodegradable six-hole plate whereas tension band wiring was done for medial malleolus. The posterior malleolus in the trimalleolar fracture (n=1) was fixed with bio-screws while the medial and lateral malleolus were fixed with metallic implants. The two cases of fracture talus were fixed with bio-screws. The two pilon fractures were stabilized by bio-implants and metallic implant combinations. The lack of confidence in the stability provided by the biodegradable pins led to supplementation with K-wires in one lateral condyle humerus [Figure 2] and two capitulum fractures. The trans-articular fixator was applied in the two cases of distal radius fracture and two cases of tibial pilon fracture. Plaster of Paris back slab was applied in the remaining 11 cases.

**Figure 3:**
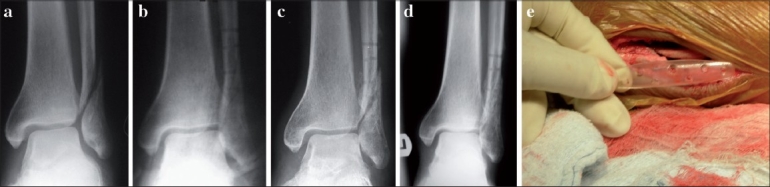
(a) Preoperative X-ray of left ankle (anteroposterior view) shows fracture lateral malleolus, (b) postoperative X-ray (anteroposterior view) of the same patient shows fracture lateral malleolus fixed with biodegradable plate, (c) follow up X-ray (anteroposterior view) of the same patient shows displaced fracture with premature weight-bearing at 5 weeks, (d) fracture united in satisfactory position, (e) intraoperative photograph shows biodegradable plate used

Complications were seen in five cases; one screw head broke while fixing a posterior malleolar fragment. Residual pain was recorded in two cases at six months follow-up. No wound healing problems were encountered (no abscess or sinus formation). One patient who walked early had mild displacement of the lateral malleolous, but the fracture healed ultimately in good position [Figure 3]. One case with distal radius fracture had loss of reduction postoperatively.

## DISCUSSION

The history of absorbable implants in the repair of bone tissue began in the late 1960s. Schmitt and Polistina^8^ (1969) first suggested the use of polyglycolide (PGA) as reinforcing pins, screws, and plates for bone surgery. Polyglycolide was hydrophilic and degraded very quickly. It lost virtually all strength within one month and all mass within six to 12 months. Adverse reactions occurred as the rate of degradation exceeded the limit of tissue tolerance, and the use of PGA alone has gradually been discontinued.^9^ A newer generation of materials is now available, created from a blend of polymers, comprising lactides, glycolides and trimethylene carbonate.^10^ These implants remain predominantly amorphous after manufacturing, which increases the bioabsorbability of these devices. However, as the mechanical properties of biodegradable implants are inferior to those of conventional metal implants,^11^ these implants have been used predominantly in fracture patterns in which internal fixation is subjected to minimal stress. Only fractures affecting the cancellous bone can be managed effectively with the array of implants currently available.^12^

The current acceptance of bio-implants in developing countries is limited by factors ranging from cost, availability and surgeon training. The first one is often prohibitive, as most patients have to buy the implants; many however ask for these, as awareness in certain sections of the society is improving due to the media and the internet. The initial cost of pins and plates is at least three to four times more than the standard AO and seven to eight times that of Indian-made implants. This acts as a significant deterrent to use, but if we consider the issues related to re-operation costs and wound complications, as well as limitations with postoperative imaging, some of the cost is offset. But as of today, the average patient is still not able to afford these implants routinely.

We also evaluated surgeon confidence in operating orthopedic sergeons, who were not familiar with their use. The plastic plates did not inspire much confidence on first usage, and the pins were often felt insufficient for primary stabilization, leading to supplementation with metal K-wires or other devices as was done in 3 of our cases. The torque needed for screw fixation is also different, and most surgeons have to go through a learning curve to find out how much force is actually enough; excessive force shears off the screw head, as was seen in one of our cases. As is the case with the new Locked Plates, perhaps a torque-limiting screwdriver may be ideal with these implants. As of now, it is our experience that the screws should be just tightened till they just touch the plate tightly, and no more.

A unique feature in plate usage is the heating bath, which softens the plate and allows molding. Comfort in the use of this is mandatory, as too much heating would destroy the implant, and too little heating is ineffective. Another issue is fracture reduction stability. If the plate is not well-molded, and fracture reduction is unstable, the force of screws being inserted could potentially move the fracture. Postoperative stability was a factor in one of our cases, who walked early, and the fracture rotated somewhat. We got a good result, but the inherent instability of the implant/bone construct towards early rotatory stress was demonstrated.

Be that as it may, long bone fractures definitely need additional stability, and external fixators in radial or pilon fractures are to be routinely recommended. As the tibial pilon injuries are high-energy injuries, some form of external fixation is needed to take the stress off the intra-articular fracture stabilization.^13^ The intra-articular part of the fracture can be easily addressed with bio-implants, with the main support coming from the external fixator. Accurate articular reduction is mandatory, and stability should be ensured; on discussing with the operating surgeons, all five felt that biopins without external fixator were not enough in distal radius or distal tibial fractures. One radius fracture displaced late during follow-up, despite the presence of biopins for the articular reduction. The consensus was that the implants were good only for intra-articular and small bone fractures; these were also adequate for moderately displaced, non-comminuted ankle fractures, where the fibula could be plated, while the medial malleolus could be fixed with screws.

The cost factor is also significant; when K-wires are compared to biopins alone, the cost is many times more, and in our scenario the benefit may not be significant.

## CONCLUSION

Biodegradable implant is an evolving modality. The use of biodegradable implant is ideally limited to intra-articular fractures and small bone injuries. Metaphyseal fracture should always be supplemented by external devices. A factor of note is surgeon comfort with the implant; a learning curve is inherent in the usage profile, and it is recommended that initial cases should be selected as well as performed after discussion with a surgeon well versed in the use of bio-implants.
